# Allotment gardening and health: a comparative survey among allotment gardeners and their neighbors without an allotment

**DOI:** 10.1186/1476-069X-9-74

**Published:** 2010-11-23

**Authors:** Agnes E van den Berg, Marijke van Winsum-Westra, Sjerp de Vries, Sonja ME van Dillen

**Affiliations:** 1 Alterra Wageningen UR, PO Box 47, NL-6700 AA Wageningen, the Netherlands; 2 Socio-Spatial Analysis group, Wageningen University, PO Box 47, NL-6700 AA Wageningen, the Netherlands; 3 Centre for Indications in Health Care (CIZ), PO Box 232, NL-3970 AE Driebergen, the Netherlands

## Abstract

**Background:**

The potential contribution of allotment gardens to a healthy and active life-style is increasingly recognized, especially for elderly populations. However, few studies have empirically examined beneficial effects of allotment gardening. In the present study the health, well-being and physical activity of older and younger allotment gardeners was compared to that of controls without an allotment.

**Methods:**

A survey was conducted among 121 members of 12 allotment sites in the Netherlands and a control group of 63 respondents without an allotment garden living next to the home addresses of allotment gardeners. The survey included five self-reported health measures (perceived general health, acute health complaints, physical constraints, chronic illnesses, and consultations with GP), four self-reported well-being measures (stress, life satisfaction, loneliness, and social contacts with friends) and one measure assessing self-reported levels of physical activity in summer. Respondents were divided into a younger and older group at the median of 62 years which equals the average retirement age in the Netherlands.

**Results:**

After adjusting for income, education level, gender, stressful life events, physical activity in winter, and access to a garden at home as covariates, both younger and older allotment gardeners reported higher levels of physical activity during the summer than neighbors in corresponding age categories. The impacts of allotment gardening on health and well-being were moderated by age. Allotment gardeners of 62 years and older scored significantly or marginally better on all measures of health and well-being than neighbors in the same age category. Health and well-being of younger allotment gardeners did not differ from younger neighbors. The greater health and well-being benefits of allotment gardening for older gardeners may be related to the finding that older allotment gardeners were more oriented towards gardening and being active, and less towards passive relaxation.

**Conclusions:**

These findings are consistent with the notion that having an allotment garden may promote an active life-style and contribute to healthy aging. However, the findings may be limited by self selection and additional research is needed to confirm and extend the current findings.

## Background

Allotment gardens originated in Europe during the 18^th ^century when plots of land were made available to poor laborers for the production of vegetables and fruits [1]. Nowadays, there are an estimated three million individual allotment gardens across Europe, which serve a variety of purposes for diverse populations [2]. Allotment gardens are a subtype of the more general category of community gardens, which include, broadly speaking, any piece of land gardened by a group of people [3]. A key characteristic of allotment gardens is that parcels of land are tended individually by plot holders and their families, contrary to shared or common types of community gardens where the overall area is tended collectively.

Allotment gardens and other types of community gardens are increasingly recognized for their potential to promote health and well-being in urban communities [4,5]. Among other things, allotment sites have been claimed to provide urban residents with opportunities to unwind from stress, interact with other members of their community, and engage in physical activity [6]. These alleged health benefits of allotment gardens receive some indirect support from epidemiological studies which have consistently shown positive relationships between urban green space and people's health and well-being [7-13]. However, it is not clear to what extent these relationships hold for allotment sites, which constitute a special kind of urban green space with a semi-public character and tight social organization. Therefore, it is important to examine health benefits of allotment gardens directly among allotment gardeners.

Thus far, empirical research among allotment gardeners has been primarily qualitative or descriptive. The findings have consistently shown that allotment gardens, like other types of community gardens, are perceived to improve general health conditions as well as to provide specific benefits related to recovery from stress, increased life satisfaction, more social contacts, and increased activity levels [14-18]. A recent field experiment among 30 allotment gardeners provides some initial evidence for the frequently cited stress-reducing effects of allotment gardening. After half an hour of gardening on the allotment, elevated salivary cortisol levels decreased by 22 percent, against a decrease of 11 percent in a control group assigned to a passive indoor reading task [19].

Although allotment sites are becoming more diverse in their ethnic and socio-demographic composition, the majority of allotment gardeners in the Netherlands and other countries are still pensioners [20-22]. The available literature suggests that allotment gardens may be especially beneficial for this older age group [23,24]. A qualitative longitudinal study in northern England describes in detail how older allotment gardeners gain a strong sense of achievement, satisfaction and aesthetic pleasure from their gardening activities. Based on these findings, the authors suggest that allotment gardens "have the potential to make a significant contribution to the healthy aging agenda" [24]. These notions are further supported by randomized intervention studies among institutionalized elderly which have found significant improvements in cognitive functioning and emotional well-being after a brief stay in the nursing home's garden [25,26].

Because research on allotment gardening has not included control groups, it is unclear whether allotment gardeners (young or old) are healthier than comparable groups without an allotment. A survey among members of a horticultural society and other social and community groups in the Midwestern United States suggests that gardening can lead to reliable differences in health and well-being. Home gardeners, the majority of whom were past retirement age, rated their overall health, physical activity, and life satisfaction higher than a matched group of non-gardeners [27]. For example, 16 percent of gardeners rated their health as excellent, compared to only 9 percent of matched non-gardeners. Recent longitudinal studies in Sweden have compared the health of leisure home owners to that of non-owners of leisure homes. These studies found that men who owned a leisure home were less likely to suffer premature departure from the paid labor force due to early retirement for health reasons [28] or early death [29]. The findings of these controlled studies among home gardeners and owners of leisure homes are of considerable interest, but it remains to be seen whether they can be generalized to allotment gardeners.

In sum, the evidence for health benefits of allotment gardens is suggestive but not sufficient to infer that allotment gardeners are healthier than comparable groups without an allotment. The main purpose of the present study was to directly compare the health, well-being and physical activity of allotment gardeners to that of controls without an allotment garden. We conducted a survey among 121 members of 12 allotment sites in the Netherlands and a control group of 63 respondents living next to the home addresses of allotment gardeners from the same sites. We hypothesized that allotment gardeners, as compared to those without an allotment, would report better health and well-being, as well as higher levels of physical activity during the summer (when the gardening season is in full swing). We also predicted that health benefits of having an allotment garden would be stronger for older respondents.

## Methods

### Study locations and respondents

The present study formed part of the "Vitamin G" research program on relationships between green space and health [30]. For this program, a research pool of eighteen allotment garden sites in and near large cities in the Netherlands was created. Previous to this study, information on these sites had been collected through visits to the sites and by means of surveys. The present study was conducted among members and neighbors of members of twelve allotment sites from the research pool, located in and around eight different cities. The surface of the sites varied between 0.75 and 25 hectare, with an average of 7 hectare. The number of members varied between 30 and 279, with a total of 1625. Six allotment sites were residential parks (with opportunities for overnight stay), four were day-recreational parks, and two were food production parks. One park was located near a highway, four were close to a railway or an airport.

Data collection lasted from the end of July until the beginning of September. Respondents could choose between the paper-and-pencil version of the survey or an online version. As an incentive, respondents were offered a chance to win one of 32 lottery tickets of 12.50 Euro. Members of the allotment organizations were invited to participate in the study by means of announcements in the news letters of the allotment organizations, which were at some sites distributed via mailboxes at the park, and at other parks were sent to the home addresses of the members. Of the eligible group of 1625 allotment garden members, 129 (8 percent) returned a complete or partially completed questionnaire. However, as the total number of allotment gardeners who noticed or read the announcement in the newsletter is unknown, the true response rate compared with that denominator cannot be provided.

The control group was selected using the addresses of allotment gardeners (from the same twelve sites included in the present study) who had participated in a previous study on garden styles [31]. A written questionnaire was sent to the two addresses closest to these addresses (200 addresses). A total of 68 persons (one per address) returned a completed or partially completed questionnaire, yielding a response rate of 34 percent in the control group. For convenience, the members of the control group will be referred to as "neighbors" throughout this article, although it should be kept in mind that they were only neighbors to allotment gardeners in a general sense, they were not neighbors in the strict sense of living next door to the specific allotment gardeners in the present sample.

Complete data on all relevant variables were available for 184 participants (121 allotment gardeners and 63 neighbors). This sample consisted of 90 men (49 percent) and 94 women (51 percent) with a mean age of 59.6 years. Table 1 provides an overview of the characteristics of the two groups.

**Table 1:** Sample characteristics

	Allotment Gardeners n = 121	Neighbors n = 63	*p*
Age (in years)	61.5 (SD 11.8; range 33-87)	55.9 (SD 13.8; range 30-85)	< .01
< 62 years	51 (42%)	42 (67%)	
*≥ *62 years	70 (58%)	21 (33%)	

Gender (male)	64 (53%)	26 (41%)	.14

Ethnicity (non-western immigrants)	4 (3%)	0 (0%)	.21

Occupation			< .05
fulltime or part-time job	43 (35%)	36 (57%)	
retired	59 (49%)	21 (33%)	
unemployed/housewife/disabled	19 (16%)	6 (10%)	

Children living at home (yes)	16 (13%)	20 (32%)	< .01

Marital status			.99
married/living together	75 (62%)	39 (62%)	
single or other	46 (38%)	24 (38%)	

Education level			.24
elementary/lower secondary	21 (17%)	6 (9%)	
higher secondary/lower vocational	46 (38%)	22 (35%)	
higher vocational/academic	54 (45%)	35 (56%)	

Income			.32
< modal	33 (27%)	18 (29%)	
modal (± 1650 euro per month)	35 (29%)	12 (19%)	
> modal	53 (44%)	33 (52%)	

Alcohol (daily users)	75 (62%)	35 (56%)	.4

Smoking (yes)	23 (19%)	12 (19%)	.99

Type of house			.06
flat or apartment	77 (64%)	30 (48%)	
semi-detached or terraced house	44 (36%)	33 (52%)	

Access to garden at home (yes)	60 (49%)	40 (64%)	.06

Living environment			.76
urban	78 (65%)	44 (70%)	
peri-urban	38 (31%)	17 (27%)	
rural	5 (4%)	2 (3%)	

Stressful life event in past year (yes)	75 (62%)	34 (54%)	.29

Physical activity in winter (days per week)	4.5 (SD 2.0)	4.3 (SD 2.1)	.49

### Dependent measures

The questionnaires for both groups included questions on respondents' health and use of health care, well-being, and physical activity. Based on these questions, 10 dependent measures were constructed.

Health and use of health care: Perceived general health was assessed by asking respondents to estimate their general health on a five-point scale, ranging from 1 = bad to 5 = excellent. This indicator originates from the SF-36 [32]. Physical constraints were assessed by the physical functioning subscale of the SF-36. Respondents were asked to indicate the extent to which their health limits them in 10 activities ranging from vigorous activities such as running to light activities such as dressing oneself (1 = not limited; 2 = a little limited, 3 = very limited). Acute health complaints were assessed by a list of 37 common, minor health problems, such as headache, coughing, sweating, and sleep problems [33]. Respondents were asked whether they had suffered from any complaint in the last 14 days. Because the number of respondents diminished with increasing numbers of complaints, the maximum number of complaints was truncated to 7. Chronic illnesses were assessed by asking respondents to indicate whether, in the year prior to the survey, they had suffered from any condition on a list of five common, life-style related types of illnesses and disorders: cardiovascular, musculoskeletal, respiratory, and mental diseases and type II diabetes (maximum number of chronic illnesses = 5). Consultations with the GP were assessed by asking respondents how often they had contacted their GP in the past 2 months (including telephone consultations).

Well-being: Stress was assessed with two items [34]. The first measured the amount of stress in the past month (from 1 = no stress to 6 = extreme stress) and the second the ability to cope with stress (from 1 = very poor, stress eats away at me to 6 = excellent, I can shake off stress easily). Because the two items were positively correlated (Cronbach's α = .63) they were combined into one stress measure by reverse-coding the ability to cope with stress and calculating the average score on the two items. Life satisfaction was assessed with the 8-item version of the Life Satisfaction Index [35]. Sample items are "My life could be happier than it is now" and "I've gotten pretty much what I expected" (1 = disagree, 2 = unsure, 3 = agree; Cronbach's α = .68). Loneliness was assessed by two items measuring the frequency of feelings of loneliness (0 = seldom/never; 1 = sometimes or frequently) and the need for social contacts (0 = no; 1 = yes) [36]. The two items were combined into a single loneliness index (range 0-2; Cronbach's α = .69). Social contacts with friends was assessed by two items measuring the frequency of contacts with friends (on a 6-point scale ranging from 1 = never to 6 = every day) and the size of one's circle of friends (1 = small, 2 = large). The two items were multiplied to obtain an index of the total amount of social contacts with friends (range 1-12).

Physical activity: Frequency of physical activity in summer and winter was measured by asking respondents to indicate, for each season, the average number of days a week on which they engaged at least half an hour in cycling, household and occupational activities, gardening, sports, and/or other intensive activities. This question was derived from the SQUASH [37]. This commonly used and well-validated Dutch questionnaire measures compliance to the guideline for physical activity in the Netherlands, which recommends people to engage at least half an hour in (at least moderately) intensive activities on 5-7 days per week [38,39]. The SQUASH comprises an aggregate and a single-item measure of physical activity. In the current study, only the single-item measure was used. Because gardening is mostly done in summer, physical activity in summer was used as the main indicator of physical activity. Physical activity in winter was used as a covariate in the analyses to control for individual differences in physical activity that are unrelated to gardening.

The dimensionality of the measures was verified by submitting the respondents' scores to a factor analysis with unrestricted factor extraction and varimax rotation. This analysis yielded a three-factor solution with 59 percent explained variance and no cross loadings greater than |.43|. The first factor included the five measures related to health and use of health care: physical constraints, chronic illnesses, acute health complaints, consultations with GP, and perceived general health (factor loadings > |.58|, eigenvalue 3.35, 28.1 percent explained variance, Cronbach's alpha .64). The second factor included the four measures related to emotional and social-well being: stress, loneliness, life satisfaction, and contacts with friends (factor loadings > |.62|, eigenvalue 1.52, 19.2 percent explained variance, Cronbach's alpha .56). The third factor consisted only of the item measuring physical activity in summer (factor loading .79, eigenvalue 1.04, 11.8 percent explained variance). These findings provide justification for a classification of measures in three dimensions related to health (including use of health care), well-being, and physical activity. For illustrative and data reduction purposes, composite indices of health and well-being were calculated as the unweighted average of the standardized scores of measures within each factor, coded so that higher scores indicate better health or well-being.

### Background variables

The questionnaire for both groups included questions about socio-demographic characteristics (gender, age, education level, household income, professional occupation, ethnicity, marital status, and having school-age children), life-style factors (smoking and drinking), and living circumstances (type of house, having access to a private or shared garden at home, and urbanity of the living environment). To control for the adverse health impacts of stressful life events, respondents were also asked to select from a predefined list whether they had experienced in the past year any stressful life event, such as marriage, death of a close one, divorce, or birth of a (grand)child (maximum number = 10; disease-related events not included to avoid overlap with health measures).

### Allotment gardening

The questionnaire for allotment gardeners contained an additional section with questions on allotment gardening, including the name of the allotment park, years of allotment gardening, type of garden (ornamental, kitchen, or mixed ornamental/kitchen garden), consumption of fresh garden produce, and the number of hours per week spent on the allotment garden in summer and winter. Allotment gardeners also estimated the percentage of time spent on five allotment activities: "gardening and maintenance", "sitting, reading and enjoying", "social activities"," administrative activities", and "other activities. Furthermore, allotment gardeners rated (on a scale from 1-5) whether they felt more or less healthy, stressed, and happy after a visit to their allotment garden, and indicated the importance of several motives for allotment gardening including health, stress relief, physical activity, and social contacts (1 = not important, 2 = a little bit important, 3 = important, 4 = very important).

### Statistical analysis

Data were analyzed with SPSS 17.0. Chi-square and Student's t-test were used to calculate and compare baseline descriptives. The data of allotment gardeners (and neighbors of the gardeners) were hierarchically nested within allotment sites. However, the intraclass correlations, computed with mixed model analyses of variance, were mostly zero or very small (≤ .06) for all dependent measures, indicating that it was not necessary to control for clustering within sites. Because the control group of neighbors did not live directly next door to the home addresses of allotment gardeners in the present study, it was also not possible nor necessary to control for dependencies between pairs of neighboring participants. Group differences in unadjusted mean scores on health, well-being and physical activity measures were analyzed using a general linear model without covariates (ANOVA). To examine impacts of allotment gardening in younger and older age groups, respondents were divided into an older and a younger group, with the average age of retirement in the Netherlands of 62 years as the cut-off point [40]. This cut-off point of 62 years also happened to represent the 50^th ^percentile of the age data. The combined impacts of allotment gardening and age on measures of health, well-being and physical activity were estimated in a covariate adjusted general linear model (ANCOVA) with allotment gardening (allotment gardeners/neighbors) and age (< 62 yrs/≥ 62 yrs) as factors and gender, education level, income, access to a garden at home, physical activity in winter, and stressful life events as covariates. Correlation tests did not show problems of multicollinearity for the covariates. Relationships between gardening characteristics and health and well-being among allotment gardeners were explored by means of supplementary linear regression analyses. Although the distributions of number of GP consults, chronic diseases, and loneliness were positively skewed, analyses of the log-transformed data yielded patterns of outcomes that were very similar to those of the untransformed data. Therefore, results are reported based on the analysis of untransformed data.

## Results

### Descriptive characteristics

Allotment gardeners owned their garden on average for 6-10 years. Fifty-four percent had an ornamental garden, 27 percent had a kitchen garden, and 19 percent had a mixed kitchen/ornamental garden. Fifty-four percent ate fresh food from the allotment regularly or more often. Allotment gardeners spent on average 32 hours per week at the allotment garden in summer, and 7 hours in winter. Allotment gardeners spent most of their time at the allotment garden on gardening and maintenance activities (62 percent). They spent 19 percent of their time on activities like sitting, reading and enjoying, 11 percent on social activities, and 5 percent on administrative activities. Eighty-four percent reported feeling healthier after a visit to the allotment garden, while 91 percent reported feeling happier and 86 percent reported feeling less stressed. Allotment gardeners rated stress relief as the most important reason for allotment gardening (56 percent rated it as very important), followed by staying active (50 percent very important), and staying healthy (42 percent very important). Social contacts were rated as very important by only 17 percent of the allotment gardeners.

Table 1 shows the socio-demographic characteristics of the allotment gardeners and the control group of neighbors. On average, allotment gardeners were more than five years older (M = 61.5 years) than the control group (M = 55.9 years). In line with their higher age, allotment gardeners were more often retired, less often had a paid job, and less often had children living at home. In both groups, the majority of respondents lived in urban areas. However, allotment gardeners more often lived in a flat or apartment (as compared to a (semi-)detached or terraced house) and they somewhat less often had access to a garden at home. Allotment gardeners did not differ from their neighbors with respect to their gender, ethnicity, marital status, levels of income and education, physical activity levels in winter, and smoking or drinking habits. In general, the allotment gardeners differed from their neighbors in dimensions that tend to be negatively related to health and well-being, in particular their higher age and less advantaged living circumstances.

Table 2 shows the unadjusted scores on composite and single measures of health, well-being and physical activity in the two groups. Despite their disadvantageous socio-demographic profile, allotment gardeners scored significantly better on physical activity in summer and composite well-being. On single measures of well-being, allotment gardeners reported significantly higher satisfaction with their lives than their neighbors. Allotment gardeners also reported marginally fewer consultations with their GP in the past two months than the control group of neighbors and somewhat fewer acute health complaints, but the group difference in composite health did not reach significance.

**Table 2:** Unadjusted means (SD) of allotment gardeners and neighbors

	Allotment gardeners *n = 121*	Neighbors *n = 63*		
***Dependent measure [range]***	***Mean*** ***(SD)***	***Mean*** ***(SD)***	***Mean Difference*** ***(95% CI)***	***p***

**Health**				

Composite z-score	0.04 (0.69)	-0.09 (0.82)	0.13 (-0.1 to 0.36)	.26

Perceived general health [1-5]	3.32 (0.79)	3.24 (0.78)	0.08 (-0.16 to 0.33)	.49

Physical constraints [1-3]	1.26 (0.32)	1.27 (0.38)	-0.01 (-0.1 to 0.11)	.89

Health complaints [0-7]	2.63 (2.32)	3.19 (2.35)	-0.56 (-1.28 to 0.15)	.12

Chronic illnesses [0-5]	0.55 (0.71)	0.54 (0.74)	0.01 (-0.21 to 0.23)	.96

GP consults past 2 months [0-6]	0.61 (0.91)	0.92 (1.34)	-0.31 (-0.64 to 0.2)	.07

**Well-being**				

Composite z-score	**0.08** **(0.67)**	**-0.14** **(0.68)**	0.22 (0.02 to 0.43	< .04

Stress [1-6]	2.53 (1.15)	2.77 (1.13)	-0.24 (-0.59 to 0.11)	.18

Life satisfaction [1-3]	**2.26** **(0.43)**	**2.12** **(0.48)**	0.14 (0 to 0.28)	< .05

Loneliness [0-2]	0.45 (0.72)	0.65 (0.85)	-0.2 (-0.43 to 0.04)	.10

Contacts with friends [1-12]	7.23 (3.04)	6.89 (2.99)	0.34 (-0.59 to 1.27)	.47

**Physical activity**				

Physical activity in summer [days p.w.]	**5.8** **(1.53)**	**4.9** **(2.15)**	0.9 (0.36 to 1.44)	< .01

### Analyses of covariance

**Figure 1:**
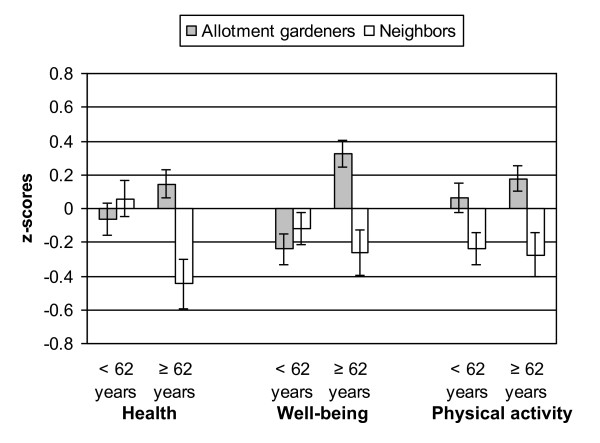
**Mean adjusted standardized scores on composite health and well-being, and physical activity in younger and older groups of allotment gardeners and neighbors**. Error bars represent ± SE. Tables

Table 3 gives an overview of the mean adjusted scores of allotment gardeners and neighbors in subsets of younger and older respondents. The outcomes are graphically illustrated in Figure 1.

**Table 3:** Adjusted means ± SE of younger and older allotment gardeners and neighbors, with test statistics.

	Allotment gardeners	Neighbors	Main effect allotment		Main effect age		Interaction age × allotment	
***Dependent measure [range]***	***Adj. Mean*** ***± SE***	***Adj. Mean*** ***± SE***	***F***	***p***	***F***	***p***	***F***	***p***

**Health**								

Composite z-score			4.67	< .04	1.68	.2	10.83	< .01

< 62 years	-0.06 ± .1	0.06 ± .1						
≥ 62 years	**0.15 ± .08**	**-0.45 ± .15**						

Perceived gen. health [1-5]			.84	.36	0.41	.53	2.91	.09

< 62 years	3.24 ± .11	3.34 ± .12						
≥ 62 years	3.37 ± .1	3.04 ± .17						

Physical constraints [1-3]			3.26	.07	15.7	< .001	11.33	< .01

< 62 years	1.23 ± .04	1.15 ± .05						
≥ 62 years	**1.27 ± .04**	1.53 ± .07*						

Health complaints [0-37]			4.01	< .05	0.38	.54	11.07	< .01

< 62 years	3.38 ± .3	2.93 ± .32						
≥ 62 years	2.04 ± .26*	**3.83 ± .45**						

Chronic illnesses [0-5]			0.4	.53	0.17	.68	4.4	< .04

< 62 years	0.64 ± .1	0.48 ± .11						
≥ 62 years	0.45 ± .09	0.76 ± .15						

GP consults [0-6]			5.4	< .03	0.07	.79	1.7	.19

< 62 years	0.69 ± .15	0.87 ± .17						
≥ 62 years	**0.52 ± .13**	**1.14 ± .23**						

**Well-being**								

Composite z-score			5.12	< .03	3.94	< .05	12.04	< .01

< 62 years	-0.24 ± .09	-0.12 ± .1						
≥ 62 years	0.32 ± .08*	**-0.26 ± .14**						

Stress [1-6]			0.18	.67	21.13	< .001	6.47	< .02

< 62 years	3.2 ± .14	2.87 ± .15						
≥ 62 years	2.05 ± .12*	2.52 ± .21						

Life satisfaction [1-3]			7.68	< .01	1.15	.29	3.54	< .06

< 62 years	2.23 ± .06	2.17 ± .07						
≥ 62 years	**2.29 ± .06**	**1.96 ± .09**						

Contacts with friends [1-12]			0.49	.49	1.94	.17	5.87	< .02

< 62 years	6.14 ± .43	7.0 ± .47						
≥ 62 years	8.07 ± .38*	6.2 ± .66						

Loneliness [0-2]			3.85	.05	0.59	.44	5.48	< .02

< 62 years	0.66 ± .11	0.62 ± .12						
≥ 62 years	0.28 ± .09*	**0.8 ± .16**						

**Physical activity**								

Physical activity in summer [days p.w.]			14.72	< .001	0.13	.72	0.59	.44

< 62 years	**5.61 ± .16**	**5.07 ± .17**						
≥ 62 years	**5.82 ± .14**	**5.0 ± .24**						

Note. Means are adjusted for income, education level, gender, life events, physical activity in winter, and having a garden at home. Means printed in bold differ per row at *p *< .05; Means of older respondents indicated with * differ per column from means of younger respondents within the same group at *p *< .05. See Table 1 for sample sizes. Df = 1, 174 for all tests.

### Health

After adjustment for income, education level, gender, life events, physical activity in winter and having a garden at home, allotment gardening had a significant positive main effect on the composite health index. On single health measures, allotment gardeners reported significantly less acute health complaints and consultations with their GP, and marginally less physical constraints. Older respondents scored somewhat lower on composite health than younger respondents, which was mainly due to a highly significant negative main effect of age on physical constraints. The predicted interaction effect between allotment gardening and age was significant or marginally significant for the composite health index and for four out of five single health measures except consultations with the GP. Allotment gardeners of 62 years and older reported a significantly better composite health than neighbors in the same age category. On the single health measures, they scored significantly better than neighbors in the same age category on physical constraints, health complaints, and GP consults, and marginally better on perceived general health and chronic illnesses. Younger allotment gardeners did not differ from younger neighbors in any health measures. Within the group of allotment gardeners, older gardeners reported significantly less health complaints than younger gardeners. In the control group of neighbors, the composite health of older respondents was marginally worse than that of younger respondents, an effect that was mainly driven by a highly significant negative impact of age on the neighbors' physical constraints.

### Well-being

After covariate adjustment, allotment gardening had a significant positive main effect on the composite index of well-being and on the single measures of life satisfaction and loneliness. Older respondents scored significantly better on composite well-being than younger respondents, which was mainly due to the fact that older respondents reported significantly less stress than younger respondents. The predicted interaction effect between allotment gardening and age was significant or marginally significant for all measures of well-being. Allotment gardeners of 62 years and older had significantly better composite well-being scores than neighbors in the same age category, and they also scored significantly or marginally better than neighbors in the same age category on all single well-being measures. Younger allotment gardeners did not differ from younger neighbors in any of the well-being measures. Within the group of allotment gardeners, composite well-being of older gardeners was significantly better than that of younger gardeners, which was due to the older gardeners reporting significantly more social contacts, less loneliness, and less stress than younger gardeners. In the group of neighbors, older and younger respondents did not differ in any of the well-being measures.

### Physical activity

After adjusting for covariates, the main positive effect of allotment gardening on physical activity in summer remained highly significant. Age did not significantly affect physical activity, neither by itself nor in interaction with allotment gardening. Older as well as younger allotment gardeners reported higher levels of physical activity than neighbors in the same age category. Additional analyses showed that 84 percent of the allotment gardeners met the Dutch guideline to engage in at least half an hour of activities on 5-7 days per week, whereas this norm was met by only 62 percent of the respondents in the control group.

### Supplementary analyses

To explore possible mechanisms underlying the greater health and well-being benefits of allotments for older respondents, we first examined whether older gardeners differed from younger gardeners in the use and experience of their allotment. The results show that, after adjustment for education level, gender, and income, allotment gardeners of 62 years and older owned their garden for a longer period of time (11-20 years) than younger gardeners (6-10 years). Older gardeners more often had a kitchen garden (38 percent) than younger gardeners (12 percent), and they more often regularly ate fresh fruits and vegetables from the allotment (64 percent) than younger gardeners (41 percent). Older gardeners spent more of their time on the allotment on gardening and maintenance (66 percent) than younger gardeners (56 percent) and less of their time on sitting, reading and enjoying (15 percent) than younger gardeners (25 percent). Older gardeners less often rated stress relief as a very important motive for gardening (46 percent) than younger gardeners (70 percent), and more often rated staying active as a very important motive for gardening (57 percent) than younger gardeners (42 percent). Thus, in general, older allotment gardeners appeared to be more oriented towards gardening and being active, and less towards passive relaxation than younger gardeners.

Next, we computed relationships between allotment gardening characteristics that differed between older and younger gardeners and the composite scores of health and well-being while controlling for age and the other covariates. These analyses revealed a significant positive relationship between well-being and the percentage of time spent on gardening and maintenance activities relative to the time spent sitting, reading and enjoying, *β *= 0.22, *t *= 3.16, *p *< .01, and a significant positive relationship between health and the motive "to stay active", *β *= 0.2, *t *= 2.15, *p *< .04. No other relationships between allotment gardening characteristics and health and well-being reached significance, *p*s > .11. These findings indicate that the greater health and well-being of older allotment gardeners may be related to their more active allotment activity patterns.

## Discussion

This study revealed that, after adjustment for socio-demographic differences, allotment gardeners reported higher levels of physical activity in summer than a control group of neighbors. Allotment gardeners also scored significantly or marginally better than the control group on several measures of health and well-being, but these differences were strongly moderated by age. Allotment gardeners of 62 years and older reported better scores on all measures of health and well-being than neighbors in the same age category, whereas younger allotment gardeners did not differ in health and well-being from younger neighbors. Taken together, these findings provide some first direct empirical evidence for health benefits of allotment gardens.

The finding that allotment gardeners generally reported higher levels of physical activity in summer than the control group of neighbors supports prevailing views of governments and other authorities that allotment gardens may contribute to achieving recommended levels of physical activity [4-6]. In summer, twelve percent more allotment gardeners than neighbors met the Dutch guideline to engage at least half an hour in at least moderately intensive activities on 5-7 days [39]. The higher self-reported activity levels of allotment gardeners are also consistent with recurrent findings of previous research that physical activity is among the primary self-reported reasons why people engage in gardening [14,15,41]. Indeed, the allotment gardeners in the present study rated being active as the second most important reason to garden. However, as much as stimulating effects of allotments on physical activity may seem intuitively plausible, nature-health research has thus far failed to demonstrate a general relationship between presence of green space and physical activity among adults [42]. This suggests that the higher physical activity levels of allotment gardeners found in the current study may be specific to allotment gardens as a special type of individually owned and maintained green space. As such, allotment gardens may provide a unique opportunity for the successful promotion of physical activity within urban communities.

The stronger health and well-being impacts of allotment gardening in the group of older respondents fit well with anecdotal and qualitative information that allotment gardens are especially beneficial for the elderly [23,24]. These findings are also in agreement with increasing experimental evidence for a causal effect of contact with gardens on the health and well-being of elderly people [25,26]. However, methodological issues may also have played a role. Most importantly, it is possible that the older allotment gardeners were self-selected for their fitness to maintain a garden, and thus, not representative of the general population of older gardeners. Such selection may have caused a "healthy gardener effect" (comparable to the "healthy worker effect" [43]) so that morbidity rates among the gardeners were underestimated. While selective loss of older, less healthy gardeners is a concern in the present study, its potential impact is somewhat limited by the strong social networks and special facilities (such as smaller plots for older gardeners) on allotment sites which support older allotment gardeners in maintaining their garden despite declining physical fitness [24]. Indeed, our sample contained quite a large proportion of older allotment gardeners (17 percent) who reported being severely disabled in one or more domains of non-vigorous physical functioning, and thus were, presumably, less fit to garden.

Alternatively, our data suggest that the greater benefits of allotment gardening for older people may be related to the fact that older gardeners use and experience their garden in a more health-supportive way than younger gardeners. Among other things, we found that older gardeners spent more of their time on the allotment on gardening as compared to passive relaxation, and we also found that this activity pattern was positively related to well-being independent of age. Conceivably, greater therapeutic benefits of gardening as compared to passive relaxation may be related to a more immersive involvement with nature and a greater sense of accomplishment and achievement from working in the garden [44]. Finally, as health and well-being may decline in old age, a pattern that was generally confirmed in our control group, it is also possible that there was more room for an influence of allotment gardening to appear among the older respondents [45]. Nevertheless, despite these plausible alternative explanations, self-selection cannot be ruled out in the present, cross-sectional study. Future longitudinal prospective or large-scale matched-pair cross-sectional studies will be needed to identify possible causal relationships of allotment gardening with health, well-being, and physical activity. In addition to analyzing the direct relationships, future research may also explore potential buffering effects of allotment gardening on negative impacts of aging [46]. The existence of such buffering effects is indicated by the moderating influence of allotment gardening on the adverse relationship between age and physical constraints found in the present study.

The present study represents only a first attempt at quantifying the benefits of allotment gardening in an objective manner. Therefore, caution is warranted in the generalization and interpretation of results. First, all findings are based on data collected through self-report and would benefit from replication with alternate behavioral and medical assessment. For example, levels of physical activity could be measured more objectively with accelerometers, stress could be measured by cortisol responses, and health could be assessed from medical records. Second, the present study focused mostly on recreational allotment sites on which food production plays only a minor role. By including more food production sites, future research may uncover additional dietary benefits of allotment gardening, preferably measured through objective registrations of food intake [47]. Third, the response rate, especially among the gardeners, was relatively small. This may have introduced the possibility of response bias, in as far as those gardeners who derived the most benefits from gardening were more likely to respond. Thus, the survey may actually overestimate health benefits of allotment gardening in the general population of allotment gardeners. Finally, the small size of the control group which, in addition, was not well matched to the group of allotment gardeners concerns another limitation that may have introduced bias and reduced the statistical power to detect possible differences. The finding that allotment gardeners did better than the control group in several domains of health, well-being and physical activity despite their disadvantageous socio-demographic profile suggests that allotment gardeners may score even better when compared to better matched control groups.

## Conclusions

The present research highlights the potential contribution of allotment gardens to an active, healthy lifestyle, especially among the elderly. Around the world, allotment gardens are increasingly under pressure from building and infrastructure developments [20,48,49]. In light of the present findings, governments and local authorities might do well to protect and enhance allotment gardens. As a case in point, Denmark has adopted special legislation that gives allotment gardens a permanent status [50]. These and related policies may help to ensure the continuation of the public health functions of allotment gardens. Indeed, allotment gardens may play a vital role in developing active and healthy living policies and programs [51,52].

## Abbreviations

ANOVA: Analysis Of VAriance; ANCOVA: Analysis of COVAriance; GP: General Practitioner; SPSS 17.0: Statistical Package for the Social Science 17; SF-36: Short-Form 36; Statistical Package for the Social Sciences version 17.0; SQUASH: Short QUestionnaire to ASses Health enhancing physical activity.

## Competing interests

The authors declare that they have no competing interests.

## Authors' contributions

AEB was the primary researcher, conceived and designed the study, participated in data collection, conducted data analysis and drafted the manuscript for publication. MWW coordinated and designed the survey and commented on drafts. SDV conceived and designed the study and commented on drafts. SMED assisted in data collection, data analysis and preparation of the first draft of the manuscript. All authors read and approved the final manuscript.
